# Comparative study of the bacterial distribution and antimicrobial susceptibility of uropathogens in older and younger patients with urinary stones

**DOI:** 10.1186/s12877-022-02886-y

**Published:** 2022-03-12

**Authors:** Jie Gu, Ping Song, Xiong Chen, Zhiming Yang, Xiaobo Zhang, Yao Bai

**Affiliations:** 1 Department of Geriatric Urology, Xiangya International Medical Center, Xiangya Hospital, Central South University, Changsha, 410008 People’s Republic of China; 2 National Clinical Research Center for Geriatric Disorders, Xiangya Hospital, Central South University, Changsha, 410008 Hunan Province People’s Republic of China; 3 Martini-Klinik Prostate Cancer Center, University Hospital Hamburg-Eppendorf, Hamburg, Germany; 4 Department of Infectious Diseases, Hunan Provincial People’s Hospital Xingsha Branch, People’s Hospital of Changsha County, Hunan Normal University, Changsha, 410008 China; 5 Urolithiasis Institute of Central South University, Changsha, 410008 People’s Republic of China; 6 Department of Urology, Hunan Provincial People’s Hospital, The first affiliated hospital of Hunan normal university, Changsha, 410005 China

**Keywords:** Urinary tract infection, Urinary calculi, Elderly, Antibiotic susceptibility, Bacterial spectrum

## Abstract

**Background:**

This study compared the bacterial spectrum and antibiotic susceptibility of uropathogens in older and younger patients with urinary stones to provide appropriate antibiotic management.

**Methods:**

We retrospectively reviewed urinary tract infection patients with urolithiasis, presented to Xiangya Hospital from March 2014 to April 2021. Patients were divided into older and younger groups according to 60 years of age. The bacterial spectrum and drug sensitivity of uropathogens were compared.

**Results:**

A total of 542 strains of uropathogens (177 in older and 365 in younger groups) were isolated from 507 patients. *E. coli* (41.8% vs 43.6%) remains the most common pathogen, followed by *E. faecalis* (6.2% vs 9.6%) in older and younger groups, respectively. Particularly, *K. pneumoniae* was significantly more frequent in older (9.6%) than in younger group (4.7%, *P* < .05). *E. faecium* was substantially more prevalent in older group (6.2%) than in younger group (2.7%, *P* < .05). The proportion of males increased in older patients (47.3%) than in younger patients (34.9%, *P* = 0.007). In both groups, major Gram-negative bacteria (*E. coli* and *K. pneumoniae*) revealed a high sensitivity over 70% to piperacillin/tazobactam, imipenem and amikacin, whereas the resistance level was high to penicillin, tetracycline and vancomycin. Major Gram-positive (*E. faecalis* and *E. faecium*) isolates demonstrated high sensitivity of over 50% to gentamicin and vancomycin in both groups. Furthermore, uropathogens isolated from younger urolithiasis patients were more susceptible to antimicrobials than those isolated from older patients.

**Conclusions:**

The male increased in the older urolithiasis patients with UTI and uropathogens microbial spectrum in older urolithiasis patients are different from younger. High susceptibility and age should be utilized in empirical antibiotic selection to avoid increased multidrug-resistant bacteria.

## Introduction

Urolithiasis is the most prevalent disease in the urinary system. Patients with urinary stones are more likely to suffer from tract obstruction dilatation and effusion, which are beneficial for pathogen reproduction and may lead to urinary tract infections (UTIs) [1]. Uropathogens can facilitate stone formation and urinary tract stones would be secondary to UTIs [2–4]. UTIs patients with urolithiasis are prone to experience more serious complications following surgery procedures, such as postoperative infection, systemic inflammation and even death due to septic shock [5, 6].

UTIs are common in older people and may cause serious complications. Symptoms may not be typical and therapeutic strategies should be adapted to this aged group, as unrevealed infection may be extremely harmful to older people but overtreatment might cause avoidable side effects and costs [7]. Improper antimicrobial drugs use and the associated rise in antimicrobial resistance have become significant health challenges [8]. Older patients with UTIs are at high risk of developing urosepsis [9]. Therefore, new empirical treatment strategies that offer safe and effective antimicrobial approaches to manage UTIs could be crucial in the older people. Studies [10, 11] have demonstrated that age (over 65 years) is the only factor for post-flexible cystoscopy UTI and that the risk of UTI increases with age.

So far, urine bacteriology and resistance patterns in older and younger patients with stones have been incompletely understood and obsolete, facilitating the demand for further knowledge to ensure efficient empirical therapies. Therefore, we conducted this study to investigate microbial spectrum and resistance patterns of uropathogens isolated from older and younger patients with urolithiasis and acquire insight into developing appropriate antimicrobial treatments.

## Patients and methods

### Patients information

This study retrospectively reviewed a total of 507 patients, presented to Xiangya hospital from March 2014 to April 2021 for seven consecutive years. These patients were diagnosed with urinary calculi with uropathogenic infection (based on urine culture). Non-enhanced CT was used to diagnose urinary calculi. Patients who previously used antibiotics within weeks, or with pregnancy, diabetes, chronic urinary retention, neurogenic bladder and immunosuppressive conditions were excluded. Urine was sampled before antibacterial treatment. We evaluated the following patient characteristics: age, gender, prevalence of UTIs, presence of UTI symptoms, stone burden (defined as the maximum diameter on CT scan), stone location, hydronephrosis, pathogen isolated and susceptibility to antibiotics.

## Urine culture

An appropriate amount of clean midstream urine was collected into a sterile container and then the specimen was used for culture-based microbiology testing. 10 μL urine sample was inoculated on blood agar and the incubation condition was 37 °C for 18–24 h (suspected fungal infection was cultured for 7 days at 28 °C). The culture will be sustained to 48 h if no microorganism growth is detected. The characteristics of the colonies (stainability and morphology) were analyzed under an inverted microscope using mass spectrometry.

### Drug sensitivity test

Microbroth dilution method was employed for the drug sensitivity and resistance tests. The MIC reference range of pathogenic bacterial colonies and standard operating procedures were determined by the Performance Standards for Antimicrobial Susceptibility [12]. A sample was regarded positive if a single micro-organism was isolated with a > 10^5^ CFU/mL concentration and associated with a > 5 leucocytes/high power field under microscopy observations.

The major reagents used in drug sensitivity test include yeast-like fungal drug-sensitive reaction strip (biomerieux Company) and a drug-sensitive reaction card (biomerieux Company) [13]. The equipment used for drug sensitivity test included an incubator (Hangzhou Lefeng Technology) and a bacterial turbidimeter (biomerieux Company).

### Statistical analysis

Continuous data were presented as mean ± SD. The chi-square test and Mann-Whitney U test were employed to compare uropathogenic distribution and susceptibility between the two groups. *P* < 0.05 was considered to indicate a statistically significant difference. SPSS 21.0 software was used for data analysis.

## Results

### Baseline characteristics of patients

**Fig. 1:**
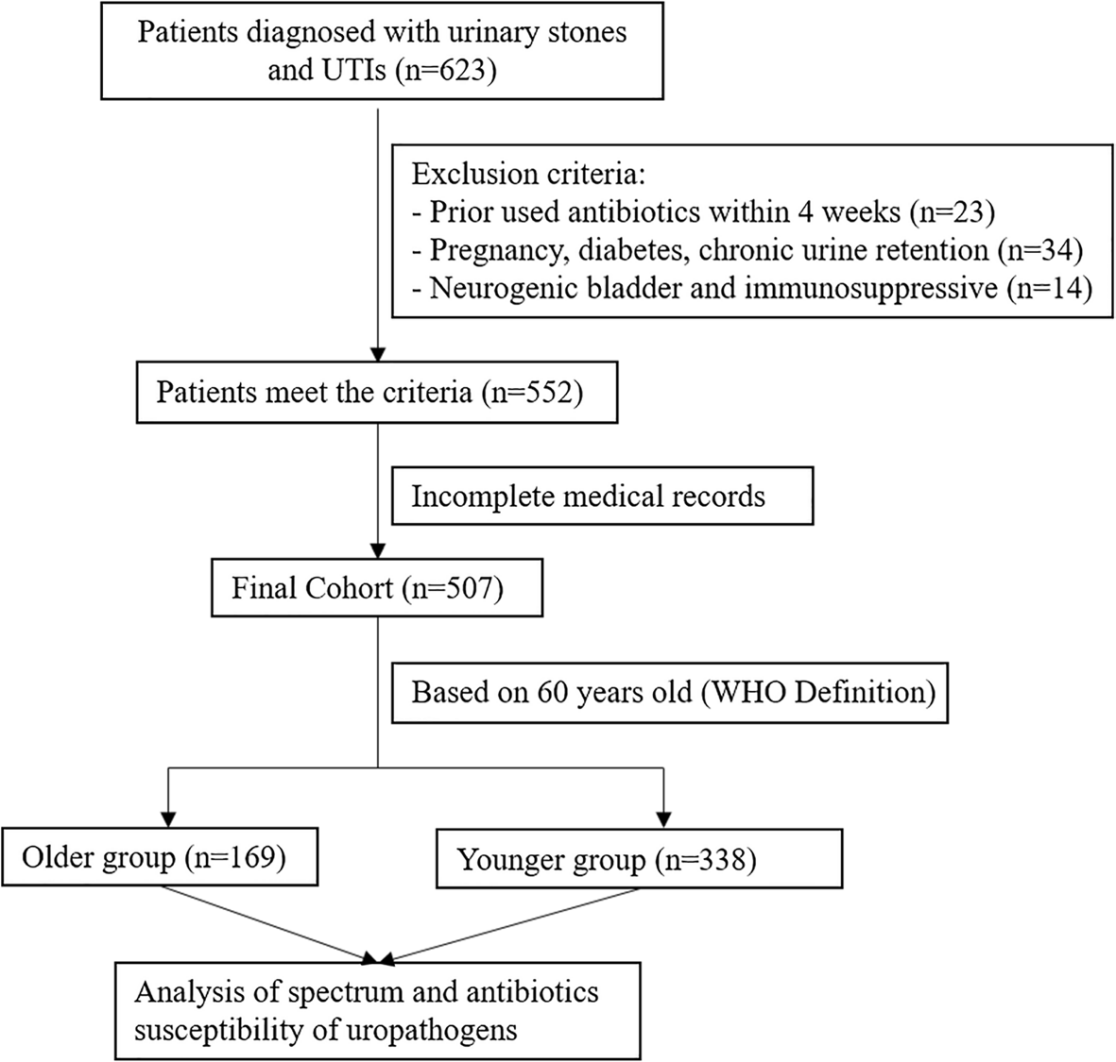
Flow diagram of patients involved in this study

**Fig. 2:**
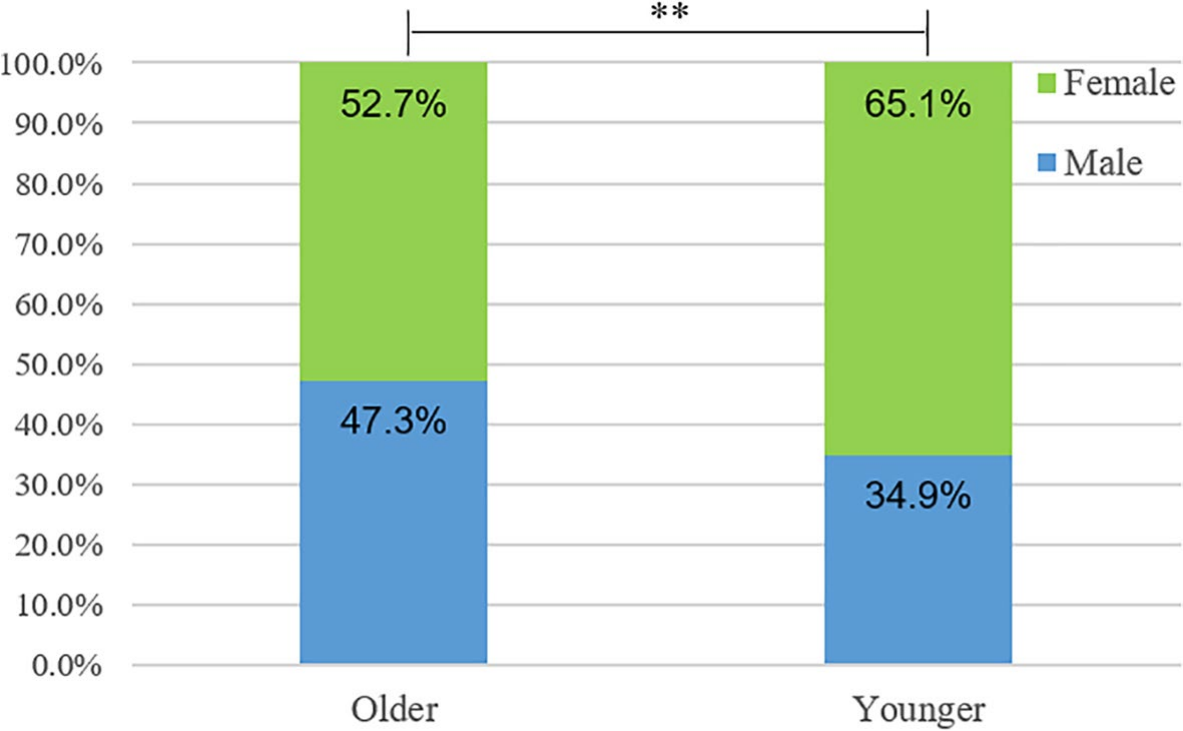
Gender trends of urinary tract infection in older and younger patients with urinary stones

The patients flow diagram was exhibited in Fig. 1 and baseline characteristics of patients were presented in Table 1. Patients were divided into two groups (older and younger) based on 60 years of age, with an average age of 67.15 ± 6.30 years and 47.42 ± 8.87 years in older and younger groups, respectively (*P* < 0.001). Significant differences in male and female distribution were observed between older and younger groups (Table 1 and Fig. 2). The pathogenic urine infection increases in males with urinary stones in older (47.3%) than in younger patients (34.9%, *P* = 0.007). In contrast, pathogenic infection in female patients is more common in younger (65.1%) than in older patients (52.7%). Overall, the most common prevalence of UTIs reported in older population with urolithiasis was cystitis 64 (37.9%) followed by asymptomatic bacteriuria 57 (33.7%), whereas the majority of the younger group was cystitis 103 (30.6%) followed by pyelonephritis 101 (29.9%) (*P* = 0.01). The presence of UTI symptoms was increased in younger patients (56.2%) compared with older patients (43.8%) (*P* < 0.001). Stone size, stone location and hydronephrosis rates did not show significant differences between the two groups. The WBC was 10.3 ± 9.8 × 10^9^/L (*n* = 83) and 9.5 ± 10.6 × 10^9^/L (*n* = 177) in older and younger groups, respectively. The C-reactive protein (CRP) was 36.7 ± 37.9 mg/L (*n* = 13) and 28.7 ± 54.2 mg/L (*n* = 30) in older group and younger groups, respectively.

**Table 1:** Patient characteristics of detected uropathogens in urinary stone patients between older and younger

	Older *N* = 169	Younger *N* = 338	*P* value
Age, y	67.15 ± 6.30	47.42 ± 8.87	*P* < 0.001
Gender			*P* = 0.007
Male, n (%)	80 (47.3%)	118 (34.9%)	
Female, n (%)	89 (52.7%)	220 (65.1%)	
Prevalence of UTIs, n (%)			*P* = 0.01
Cystitis	64 (37.9%)	103 (30.6%)	
Asymptomatic bacteriuria	57 (33.7%)	85 (25.2%)	
Pyelonephritis	28 (16.6%)	101 (29.9%)	
Urosepsis	3 (1.8%)	5 (1.5%)	
Other	17 (10.1%)	42 (12.5%)	
Presence of UTI symptoms, n (%)	74 (43.8%)	190 (56.2%)	*P* < 0.001
Stone burden (mm)	22 ± 4.8	21 ± 8.1	*P* = 0.259
Stone Location			*P* = 0.461
Kidney, n (%)	83 (49.2%)	179 (53.0%)	
Ureteral, n (%)	49 (28.8%)	93 (27.5%)	
Bladder, n (%)	6 (3.5%)	9 (2.7%)	
Multiple, n (%)	31 (18.5%)	57 (16.9%)	
Hydronephrosis, n (%)	72 (42.6%)	160 (47.3%)	*P* = 0.313

Chi-square test was performed to detect differences in patient characteristics

## Characters of distribution of Uropathogenic organisms

**Fig. 3:**
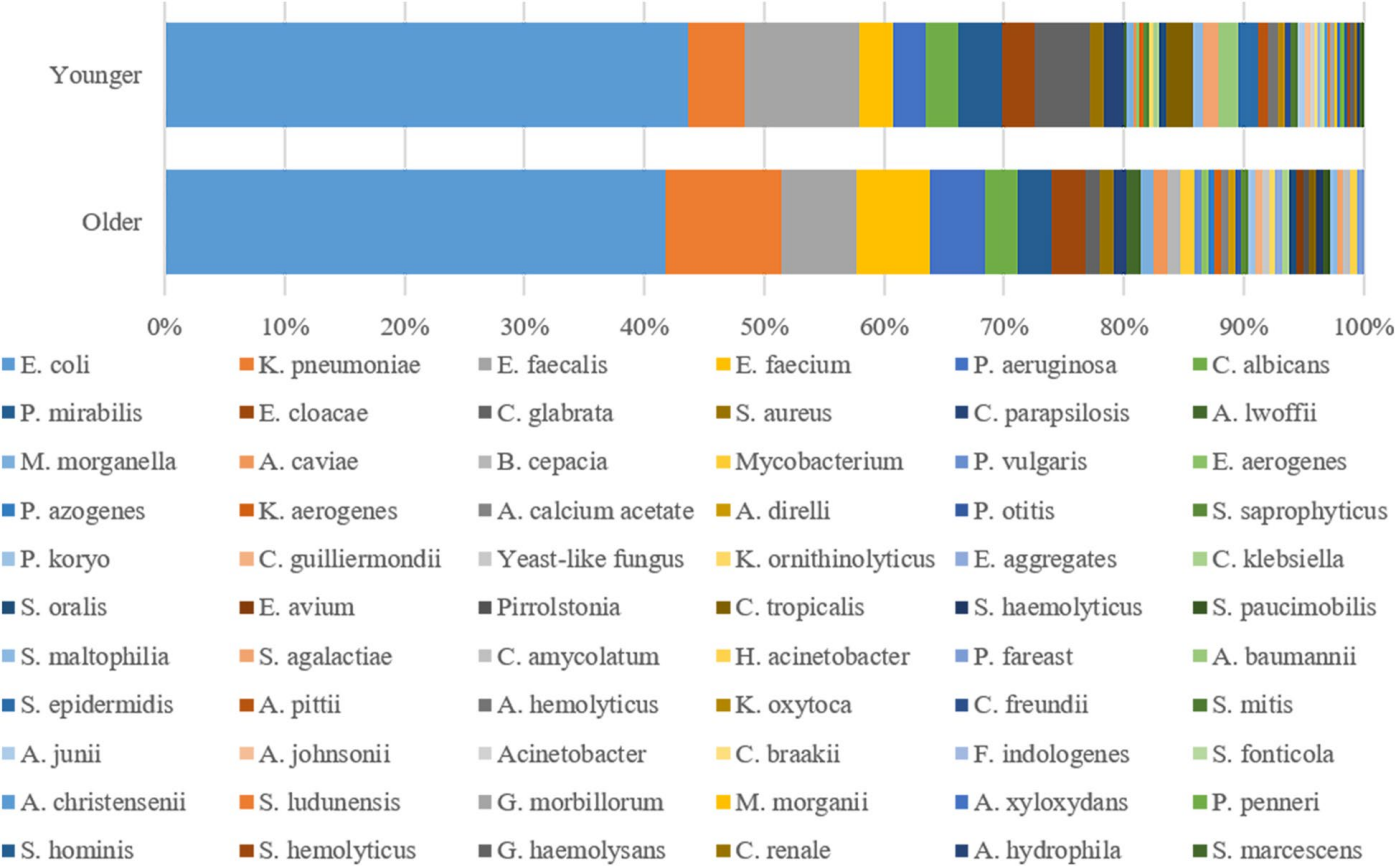
Distribution of uropathogens isolated from (**A**) older and (**B**) younger patients with urinary stones

**Fig. 4:**
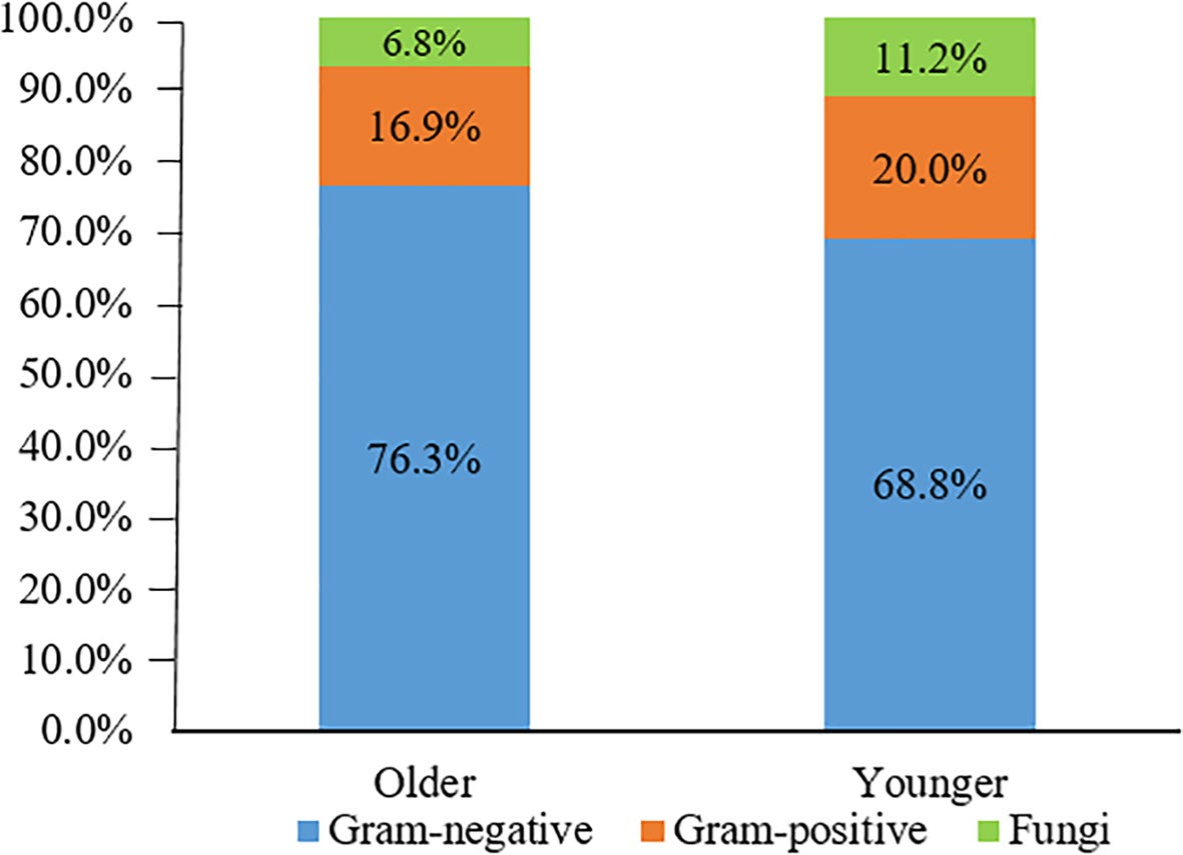
Uropathogens detection rates in older and younger patients with urinary stones

Figure 3 depicts the types and proportions of uropathogens. A total of 41 bacterial types were isolated from 170 older patients and 49 bacterial types from 337 younger patients. Figure 4 illustrates the detection rates of uropathogens in older and younger patients with urolithiasis, and Gram-negative bacteria were the most detectable category in both groups (76.3% vs 68.8%).

Significant differences were found in the bacterial spectrum of older and younger patients (Table 2). In particular, *K. pneumoniae* was significantly more frequent in older (9.6%) than in younger patients (4.7%, *P* < .05). Major Gram-positive bacterial *E. faecium* was observed to be more prevalent in oldergroup (6.2%) than in younger group (2.7%, *P* < .05). *E. coli* dominated in both older (41.8%) and younger groups (43.6%). *E. faecalis* was the second most common uropathogen inferior only to *E. coli* in total. Furthermore, the incidence of Candida glabrata infection was higher in older group (4.7%) than in younger group (1.1%, *P* < .05). Among 542 strains, 150 (84.7%) and 316 (86.5%) strains belonged to the top 15 most frequent pathogens in older and younger groups, respectively. Among Enterobacteriaceae uropathogens, extended-spectrum β-lactamase (ESBL) producers identified no differences between elderly and non-elderly groups in *E. coli* and K. pneumonia.

**Table 2:** Comparison of top 15 most frequently detected uropathogens in urinary stone patients between older and younger

Older	N (%) ***N*** = 177	P	Younger	N (%) *N* = 365	P
*Escherichia Coli*^n^	74 (41.8)	.699	*Escherichia Coli*^n^	159 (43.6)	.699
*E. coli* ESBL (+)	45 (60.8)	.806	*E. coli* ESBL (+)	94(59.1)	.806
*E. coli* ESBL (−)	29 (39.2)		*E. coli* ESBL (−)	65(40.9)	
*Klebsiella pneumoniae*^n^	17 (9.6)	.026*	*Enterococcus faecalis*^p^	35 (9.6)	.186
K. pneumonia ESBL(+)	3 (17.6)	.244	*Klebsiella pneumoniae*^n^	17 (4.7)	.026*
*K. pneumoniae* ESBL(−)	14 (82.4)		*K. pneumoniae* ESBL(+)	6 (35.3)	.244
*Enterococcus faecalis*^p^	11 (6.2)	.186	*K. pneumoniae* ESBL(−)	11 (64.7)	
*Enterococcus faecium*^p^	11 (6.2)	.049*	Candida glabrata^F^	17 (4.7)	.036*
*Pseudomonas aeruginosa*^n^	8 (4.5)	.278	*Proteus mirabilis*^n^	13 (3.6)	.653
Candida albicans^F^	5 (2.8)	.955	Candida albicans^F^	10 (2.7)	.955
*Proteus mirabilis*^n^	5 (2.8)	.653	*Enterococcus faecium*^p^	10 (2.7)	.049*
Enterobacter cloacae^N^	4 (2.3)	.741	*Pseudomonas aeruginosa*^n^	10 (2.7)	.278
Candida glabrata^F^	2 (1.1)	.036*	Enterobacter cloacae^N^	10 (2.7)	.741
*Staphylococcus aureus*^p^	2 (1.1)	.972	Candida tropicalis^F^	8 (2.2)	.165
Candida parapsilosis^F^	2 (1.1)	.642	Acinetobacter baumannii^N^	6 (1.6)	0.047*
Acinetobacter lwoffii^N^	2 (1.1)	.208	S. epidermidis^P^	6 (1.6)	0.047*
Morgan Morganella^N^	2 (1.1)	.458	Candida parapsilosis^F^	6 (1.6)	.642
Aeromonas caviae^N^	2 (1.1)	.208	Streptococcus agalactiae^P^	5 (1.4)	.401
Burkholderia cepacia^N^	2 (1.1)	.042	*Staphylococcus aureus*^p^	4 (1.4)	.972

Chi-square test was performed to detect differences in specific uropathogens distribution. *P < 0.05 illustrates statistical significance. ^N^ Gram-negative, ^P^ Gram-positive, ^F^ Fungi

## Phenotypic analysis for drug susceptibility of urinary pathogen

### The susceptibility of major gram-negative bacteria (*E. coli* and *K. pneumoniae*) to antimicrobial drugs

*E. coli* demonstrated a higher susceptibility to levofloxacin and ciprofloxacin (*P* < .05) in younger than older patients. *E. coli* bacteria was observed over 60% sensitivity to ceftazidime, cefoperazone/sulbactam, cefotetan, piperacillin/tazobactam, imipenem, nitrofurantoin and amikacin in older and younger groups, whereas the resistance level was high in both groups to penicillin, tetracycline, vancomycin and ampicillin. Cefotetan and imipenem presented a higher susceptibility to K. pneumonia in younger than older patients (*P* < .05). K. pneumonia bacteria exhibited more than 60% sensitivity to cefoperazone/sulbactam, gentamicin, tobramycin, piperacillin/tazobactam, aztreonam, imipenem and amikacin in older and younger patients. In contrast, the resistance level was high in both groups to penicillin, tetracycline, vancomycin, ampicillin, and nitrofurantoin (Table 3). Higher overall susceptibility trends were observed in younger than older patients (Mann–Whitney U test) regarding the major Gram-negative bacteria *E. coli* (*P* < .05).

**Table 3:** The susceptibility of main Gram-Negative bacteria to common antibiotics (%)

Antibiotics	*E. coli*		*K. pneumoniae*	
	Older	Younger	Older	Younger
Ceftriaxone	31.1%	33.3%	58.8%	58.8%
Cefazolin	25.7%	25.8%	52.9%	29.4%
Cefpidoxime	12.2%	19.5%	23.5%	23.5%
Ceftazidime	60.8%	64.8%	58.8%	76.5%
Cefoperazone/Sulbactam	70.3%	79.2%	70.6%	82.4%
Cefotetan	63.5%	67.9%	47.1%*	82.4%*
Penicillin	0.0%	0.6%	0.0%	0.0%
Tetracycline	0.0%	0.0%	0.0%	0.0%
Gentamicin	47.3%	56.6%	64.7%	70.6%
Tobramycin	54.1%	56.6%	64.7%	64.7%
Vancomycin	0.0%	0.6%	0.0%	0.0%
Ampicillin	9.5%	10.7%	0.0%	0.0%
Piperacillin/ Tazobactam	81.1%	86.8%	70.6%	82.4%
Aztreonam	48.6%	54.1%	64.7%	76.5%
Imipenem	90.5%	87.4%	70.6%*	94.1%*
Meropenem	62.2%	52.2%	58.8%	41.2%
Compound trimethoprim	44.6%	53.5%	76.5%	70.6%
Nitrofurantoin	78.4%	75.5%	5.9%	5.9%
Levofloxacin	14.9%*	32.7%*	41.2%	70.6%
Amikacin	87.8%	91.2%	82.4%	88.2%
Ciprofloxacin	14.9%*	30.8%*	29.4%	58.8%

Chi-square test was performed to detect differences in specific uropathogens distribution. *P < 0.05 illustrates statistical significance

### The susceptibility of the major gram-positive bacteria (*E. faecalis* and *E. faecium*) to antibiotics

Table 4 illustrates the susceptible rates of major Gram-positive bacteria (*E. faecalis* and *E. faecium*) to various antibiotics. Overall, major Gram-positive *E. faecalis* bacteria exhibited significant differences in overall susceptibility trends between older and younger patients (P < .05, Mann–Whitney U test). Gentamicin and tobramycin revealed a high level of in vitro susceptibility against *E. faecalis* and *E. faecium*, whereas cephalosporins indicated relatively high resistance rates. Nitrofurantoin exhibited 80.0% susceptibility in younger patients but 63.6% in older patients to *E. faecalis*. Gentamicin confirmed a high susceptibility rate of 81.8% in older patients and only 50% susceptibility was observed in younger patients to *E. faecium*.

**Table 4:** The susceptibility of main Gram-Positive bacteria to common antibacterial drugs (%)

Antibiotics	*Enterococcus faecalis*		*Enterococcus faecium*	
	Older	Younger	Older	Younger
Cefoperazone/Sulbactam	9.1%	14.3%	0.0%	10.0%
Penicillin	81.8%	71.4%	9.1%	0.0%
Tetracycline	0.0%	8.6%	18.2%	40.0%
Gentamicin	54.5%	62.9%	81.8%	50.0%
Tobramycin	0.0%	2.9%	0.0%	0.0%
Vancomycin	90.9%	85.7%	100.0%	100.0%
Ampicillin	81.8%	80.0%	9.1%	10.0%
Piperacillin/Tazobactam	9.1%	11.4%	0.0%	0.0%
Aztreonam	0.0%	5.7%	0.0%	0.0%
Imipenem	9.1%	8.6%	0.0%	0.0%
Meropenem	9.1%	0.0%	0.0%	0.0%
Compound trimethoprim	0.0%	8.6%	0.0%	0.0%
Nitrofurantoin	63.6%	80.0%	9.1%	30.0%
Levofloxacin	72.7%	77.1%	9.1%	40.0%
Amikacin	9.1%	11.4%	0.0%	0.0%
Ciprofloxacin	54.5%	62.9%	9.1%	10.0%

Chi-square test was performed to detect differences in specific uropathogens distribution. *P < 0.05 illustrates statistical significance

## Discussion

In this study, our results revealed significant uropathogen distribution differences between older and younger groups according to 60 years of age (WHO Definition of Aging) [14]. Pathogenic urine infection is increased in males with urinary stones in older patients (47.3%) than in younger patients (34.9%, *P* < .001). In contrast, the pathogenic infection of female patients is more common in younger (65.1%) than in older patients (52.7%). Furthermore, our study revealed that major Gram-positive bacterial *E. coli* and major Gram-negative bacterial *E. faecalis* exhibited higher susceptibility trends isolated from younger patients than isolated from older patients. To our knowledge, this is the first study to investigate bacterial spectrum of uropathogenic and antibiotic susceptibility in older and younger patients with stones.

In both groups, *E. coli* was the most common uropathogen in UTIs patients with urolithiasis, which is consistent with a previous study [15]. However, prior research revealed that less than 62–75% of uncomplicated urinary tract infections were reported [16, 17]. This discrepancy could be explained by more complex bacterial patterns in stone patients which were mainly reflected in the presence of calculi, invasive procedures, catheter-associated placement, etc. [18].

*E. faecalis* and K. pneumonia were the second most frequent uropathogens in younger and older patients with stones, respectively. *E. faecalis* has been demonstrated to cause healthcare-associated infections and display resistance to various broad-spectrum antibiotics by acquisition of resistance traits as well as the ability to form biofilms [19]. The matrix and surface of stone provide a good foundation and adhesion for bacterial growth and reproduction, eventually resulting in chronic bacteriuria establishment [20].

The bacterial spectrum of uropathogens isolated from older urinary stone patients differs from younger patients (*P* < .05). *E. faecium* and *Pseudomonas aeruginosa* occupy the fourth and fifth positions in the older group, respectively. Meanwhile, Candida glabrata and *Proteus mirabilis* are ranked fourth and fifth in the younger group. The bacterial spectrum difference between older and younger groups may due to the fact that elderly exhibiting lower immunity power and more basic disease [21]. Risk factors for UTI in the elderly differ from those in the younger population. Factors that increase the possibilities of forming UTIs include age-related changes in immune function (immunosenescence), nosocomial pathogen exposure, and a higher number of comorbidities [21, 22]. Additionally, a previous study showed that age over 65 was the only risk factor present in all patients with urinary tract infection development following flexible cystoscopy [11].

Interestingly, we found that gender distribution in UTIs patients with urinary stones is significantly different between the two groups. Females (220, 65.1%) account for a higher proportion in younger group, which may be attributed to the fact that women with short urethral than men in anatomy and younger women may engage in sexual activity more frequently [10, 23]. However, the proportion of males increased from 118 (34.9%) in younger group to 80 (47.3%) in older group (*P* < .01). A possible reason was that older men typically exhibit urodynamic dysfunction owing to prostatic hypertrophy. In addition, uncircumcised clearly increases the risk of UTIs in older men [24–26] Various chronic diseases in older males (diabetes, spinal cord injury, indwelling, or intermittent bladder catheterization) also contribute to UTIs development [23]. As we observed an increased percentage of females in younger group, the possibility would be that sexual activity can transport germs from the vagina to the urethra then resulting in UTIs. A previous study showed that prostatitis could cause recurrent UTIs [27].

Uropathogens showed higher susceptibility trends isolated from younger patients than older patients (*P* < .05). These differences were more pronounced for main Gram-negative bacteria K. pneumonia, and specifically for the antibiotics, cefotetan and imipenem, which were more sensitive to younger UTIs patients with urinary stones than older patients (*P* < .05). Older patients may have lower susceptibility rates since they are more frequently hospitalized, as a result of the increased average life expectancy, weak immune system, and recurring infections. Hospitalizations are known to increase the transmission of bacterial strains between hospitals and the community [28]. Overall, antimicrobial sensitivity and resistance profiles indicate that empirical antibiotic selection should take the patient’s age into consideration.

In our study, the susceptibility of E.coli and Klebsiella to ceftriaxone and levofloxacin was low compared to community-acquired UTIs [4, 8]. Even meropenem also exhibits low susceptibility. Ceftriaxone and levofloxacin resistance may be partly explained by the increasing quinolone resistance and ESBL, AmpC, and carbapenemases, such as K.pneumoniae carbapenemase or metallo-β-lactamase [29]. A multicenter study conducted in seven countries revealed regional differences in microbial isolates and susceptibility [4]. According to a nosocomial infections surveillance system report, susceptibility rates of *E. coli* isolates to cefotaxime and levofloxacin dramatically decreased from 2007 to 2017 [30]. A study in China reported that the uropathogens exhibited marked multidrug resistance and a large scale of the uropathogens produce β-lactamase [2]. Interestingly, one similar research in our region showed that drug resistance rates remained high for commonly used drugs, and local antibiogram patterns should be considered before initiating empiric antibiotic therapy for UTI patients with urolithiasis [15]. What’s more, this distinction might be because patients with stones are more likely to suffer from invasive procedures, catheter-associated placement, etc.

In general, overuse and misuse of antimicrobials have contributed tocontinued resistance development, posing a serious public health burden [31, 32]. A typical example is the methicillin-resistant *Staphylococcus aureus*, which is responsible for considerable difficult-to-treat infections in humans [33]. The increasing antimicrobial resistance of uropathogens is challenging the paradigm of empirical antibiotic therapy for UTIs patients with urolithiasis, underlining the need for developing appropriate treatment strategies. New strategies with safe and effective approaches to manage UTIs with stones could have an important role in the older people.

One of the limitations of our study is that uropathogens isolated from upper and lower urinary stones were not distinguished and compared. Furthermore, it is preferable to combine stone culture results (even blood culture if necessary), as midstream urine cultures might not completely reflect the reality of UTIs. Moreover, the effects of high susceptibility and resistant antibiotics after the patients were treated needed to be evaluated in the future. Given that not all patients underwent surgery, we do not have a complete stone composition. Given the retrospective study design, it is possible that there are clinical parameters related to the treatment decisions and clinical outcomes we did not include or miss. Additionally, we excluded a significant number of patients with diabetes, chronic urinary retention and incomplete medical records, which may limit the impact and generalizability of our findings.

## Conclusions

The ratio of male patients increased in the older urolithiasis patients and uropathogenic microbial spectrum is different in older urolithiasis patients from younger patients. Uropathogens isolated from younger urolithiasis patients were more susceptible to antimicrobials than older patients. The uropathogens isolated from patients with stones revealed marked multidrug resistance in both groups. The empirical use of antibiotics with low resistance rates should be reserved and age should be considered to avoid the increase of multidrug-resistant bacteria.

## Data Availability

The dataset generated during and/or analyzed during the current study is available from the corresponding author on reasonable request.

## Abbreviations

*E. coli*: *Escherichia Coli*
*E. faecalis*: *Enterococcus faecalis*
*K. pneumoniae*: *Klebsiella pneumoniae*
*E. faecium*: *Enterococcus faecium*
UTIs: Urinary tract infections
ESBL: Extended-spectrum β-lactamse
CRP: C-reactive protein

## References

[1] Miano R, Germani S, Vespasiani G. Stones and urinary tract infections. Urol Int. 2007; 10.1159/000104439. 10.1159/000104439 17726350

[2] Chen D, Zhang Y, Huang J, Liang X, Zeng T, Lan C, et al. The analysis of microbial spectrum and antibiotic resistance of uropathogens isolated from patients with urinary stones. Int J Clin Pract. 2018; 10.1111/ijcp.13205. 10.1111/ijcp.13205 29790623

[3] Zanetti G, Paparella S, Trinchieri A, Prezioso D, Rocco F, Naber KG (2008). Infections and urolithiasis: current clinical evidence in prophylaxis and antibiotic therapy. Archivio Italiano di Urologia e Andrologia.

[4] De Lorenzis E, Alba AB, Cepeda M, Galan JA, Geavlete P, Giannakopoulos S, et al. Bacterial spectrum and antibiotic resistance of urinary tract infections in patients treated for upper urinary tract calculi: a multicenter analysis. Eur J Clin Microbiol Infect Dis. 2020; 10.1007/s10096-020-03947-z. 10.1007/s10096-020-03947-z 32557326

[5] Erdil T, Bostanci Y, Ozden E, Atac F, Yakupoglu YK, Yilmaz AF, et al. Risk factors for systemic inflammatory response syndrome following percutaneous nephrolithotomy. Urol Res. 2013; 10.1007/s00240-013-0570-y. 10.1007/s00240-013-0570-y 23712738

[6] Soriano A, Mansour R, Horovitz Y (2014). Bacterial endocarditis following lithotripsy: an unusual complication caused by a non-invasive procedure. Isr Med Assoc J.

[7] Beyer I, Mergam A, Benoit F, Theunissen C, Pepersack T. Management of urinary tract infections in the elderly. Z Gerontol Geriatr. 2001; 10.1007/s003910170080. 10.1007/s003910170080 11393008

[8] Tandogdu Z, Kakariadis ETA, Naber K, Wagenlehner F, Johansen TEB. Appropriate empiric antibiotic choices in health care associated urinary tract infections in urology departments in Europe from 2006 to 2015: a Bayesian analytical approach applied in a surveillance study. PLoS One. 2019; 10.1371/journal.pone.0214710. 10.1371/journal.pone.0214710 PMC6483335 31022187

[9] Chin BS, Kim MS, Han SH, Shin SY, Choi HK, Chae YT, et al. Risk factors of all-cause in-hospital mortality among Korean elderly bacteremic urinary tract infection (UTI) patients. Arch Gerontol Geriatr. 2011; 10.1016/j.archger.2010.05.011. 10.1016/j.archger.2010.05.011 20579748

[10] Foxman B. Urinary tract infection syndromes. Occurrence, recurrence, bacteriology, risk factors, and disease burden. Infect Dis Clin N Am. 2014; 10.1016/j.idc.2013.09.003. 10.1016/j.idc.2013.09.003 24484571

[11] Cusumano JA, Hermenau M, Gaitanis M, Travis M, LaPlante KL, Tran TY, et al. Evaluation of post–flexible cystoscopy urinary tract infection rates. Am J Heal Pharm. 2020; 10.1093/ajhp/zxaa270. 10.1093/ajhp/zxaa270 32827037

[12] CLSI. Performance standards for antimicrobial susceptibility testing performance standards for antimicrobial susceptibility testing suggested citation. CLSI Doc M02-A11. 2018:1–13.

[13] van Belkum A, Bachmann TT, Lüdke G, Lisby JG, Kahlmeter G, Mohess A, et al. Developmental roadmap for antimicrobial susceptibility testing systems. Nat Rev Microbiol. 2019. 10.1038/s41579-018-0098-9. 10.1038/s41579-018-0098-9 PMC7138758 30333569

[14] WHO. Decade of healthy ageing. World heal. Organ. 2020:1–24. https://www.who.int/initiatives/decade-of-healthy-ageing. Accessed 2021.

[15] Bai Y, Liu Q, Gu J, Zhang X, Hu S. Analysis of urinary pathogen cultures and drug sensitivity in patients with urinary stones for five consecutive years in Xiangya hospital, China. Infect Drug Resist. 2020; 10.2147/IDR.S241036. 10.2147/IDR.S241036 PMC7227811 32494167

[16] Adapala RR, Shetty R, Venugopal P, Prabhu GGL, Yalla D, Unnikrishnan B. Renal salvage, an achievable goal in patients with emphysematous pyelonephritis: outcomes of an algorithmic renal preserving strategy. Urol Ann. 2020; 10.4103/UA.UA_67_19. 10.4103/UA.UA_67_19 PMC7292438 32565654

[17] Adegun PT, Odimayo MS, Olaogun JG, Emmanuel EE. Comparison of uropathogens and antibiotic susceptibility patterns in catheterized ambulant middle-aged and elderly nigerian patients with bladder outlet obstruction. Turk J Urol. 2019; 10.5152/tud.2018.25588. 10.5152/tud.2018.25588 PMC6342574 29975632

[18] Flores-Mireles AL, Walker JN, Caparon M, Hultgren SJ. Urinary tract infections: epidemiology, mechanisms of infection and treatment options. Nat Rev Microbiol. 2015; 10.1038/nrmicro3432. 10.1038/nrmicro3432 PMC4457377 25853778

[19] Parthasarathy S, Jordan LD, Schwarting N, Woods MA, Abdullahi Z, Varahan S, et al. Involvement of chromosomally encoded homologs of the RRNPP protein family in enterococcus faecalis biofilm formation and urinary tract infection pathogenesis. J Bacteriol. 2020; 10.1128/JB.00063-20. 10.1128/JB.00063-20 PMC7417834 32540933

[20] Nickel JC, Emtage J, Costerton JW. Ultrastructural microbial ecology of infection-induced urinary stones. J Urol. 1985; 10.1016/s0022-5347(17)49116-6. 10.1016/s0022-5347(17)49116-6 3981713

[21] Pawelec G, Larbi A. Immunity and ageing in man: annual review 2006/2007. Exp Gerontol. 2008; 10.1016/j.exger.2007.09.009. 10.1016/j.exger.2007.09.009 17977683

[22] Rowe TA, Juthani-Mehta M. Urinary tract infection in older adults. Aging Health. 2013;9(5). 10.2217/ahe.13.38. 10.2217/ahe.13.38 PMC3878051 24391677

[23] Foxman B. Epidemiology of urinary tract infections: incidence, morbidity, and economic costs. Disease-a-Month. 2003; 10.1067/mda.2003.7. 10.1067/mda.2003.7 12601337

[24] Wiswell TE, Hachey WE. Urinary tract infections and the uncircumcised state: an update. Clin Pediatr (Phila). 1993; 10.1177/000992289303200301. 10.1177/000992289303200301 8453827

[25] To T, Agha M, Dick PT, Feldman W. Cohort study on circumcision of newborn boys and subsequent risk of urinary-tract infection. Lancet. 1998; 10.1016/s0022-5347(05)68380-2. 10.1016/S0140-6736(98)02392-7 9851381

[26] Wiswell TE, Smith FR, Bass JW (1985). Decreased incidence of urinary tract infections in circumcised male infants. Pediatrics..

[27] Lipsky BA. Prostatitis and urinary tract infection in men: What’s new; what’s true? Am J Med. 1999; 10.1016/s0002-9343(99)00017-0. 10.1016/s0002-9343(99)00017-0 10190383

[28] Ott E, Saathoff S, Graf K, Schwab F, Chaberny IF. The prevalence of nosocomial and community acquired infections in a university hospital: an observational study. Dtsch Arztebl Int. 2013. 10.3238/arztebl.2013.0533. 10.3238/arztebl.2013.0533 PMC3782020 24069074

[29] Bush K. Past and present perspectives on β-lactamases. Antimicrob Agents Chemother. 2018; 10.1128/AAC.01076-18. 10.1128/AAC.01076-18 PMC6153792 30061284

[30] JANIS, Annual Open Report 2020 (all facilities), Japan Nosocomial Infections Surveillance (CLSI2012 Version), Clinical Laboratory Division. 2020:1–37. https://janis.mhlw.go.jp/english/report/open_report/2020/3/1/ken_Open_Report_Eng_202000_clsi2012.pdf.

[31] Hulscher ME, Grol RP, van der Meer JW. Antibiotic prescribing in hospitals: a social and behavioural scientific approach. Lancet Infect Dis. 2010; 10.1016/S1473-3099(10)70027-X. 10.1016/S1473-3099(10)70027-X 20185095

[32] Bell BG, Schellevis F, Stobberingh E, Goossens H, Pringle M. A systematic review and meta-analysis of the effects of antibiotic consumption on antibiotic resistance. BMC Infect Dis. 2014; 10.1186/1471-2334-14-13. 10.1186/1471-2334-14-13 PMC3897982 24405683

[33] Chambers HF, DeLeo FR. Waves of resistance: Staphylococcus aureus in the antibiotic era. Nat Rev Microbiol. 2009; 10.1038/nrmicro2200. 10.1038/nrmicro2200 PMC2871281 19680247

